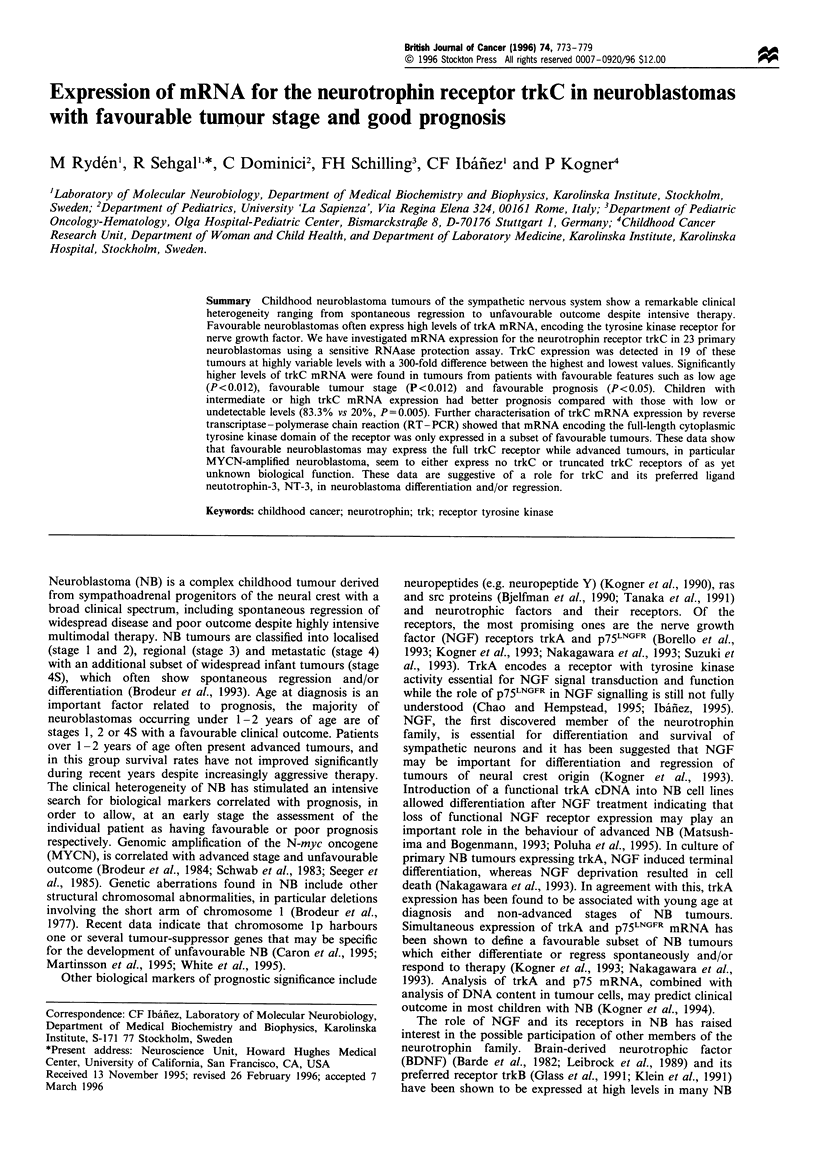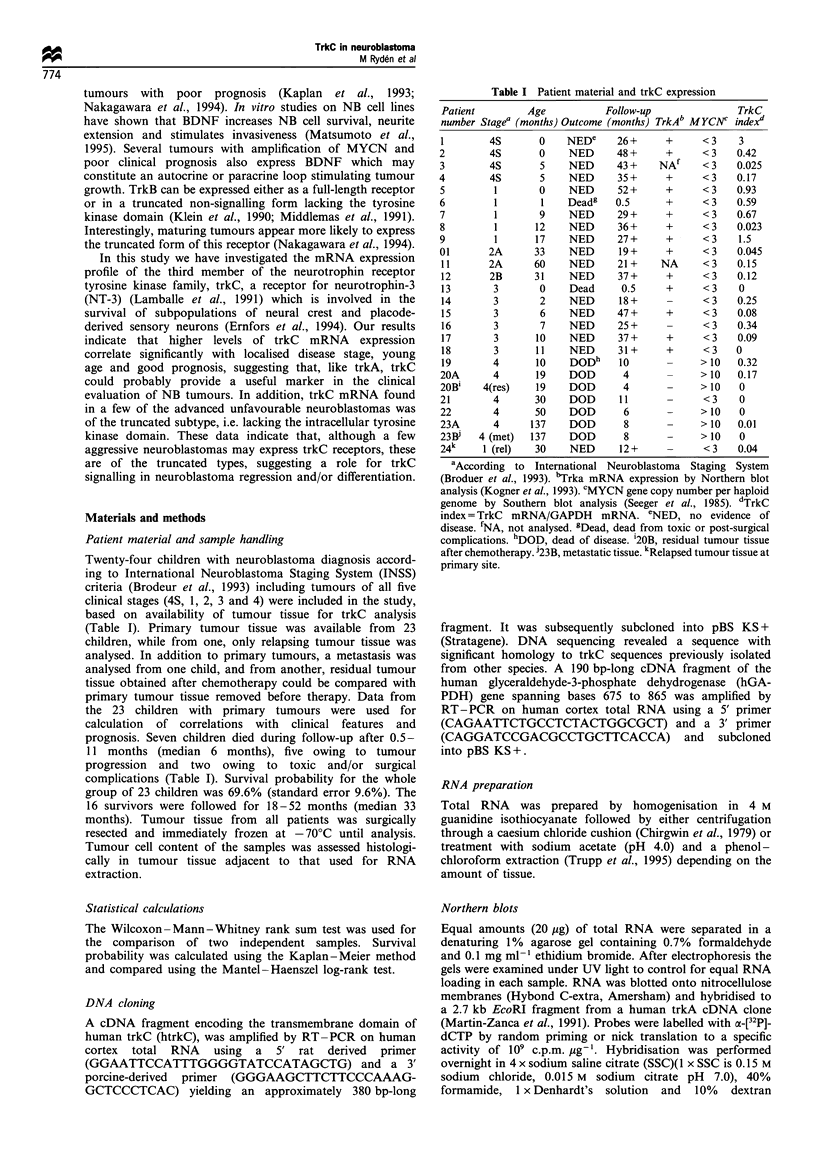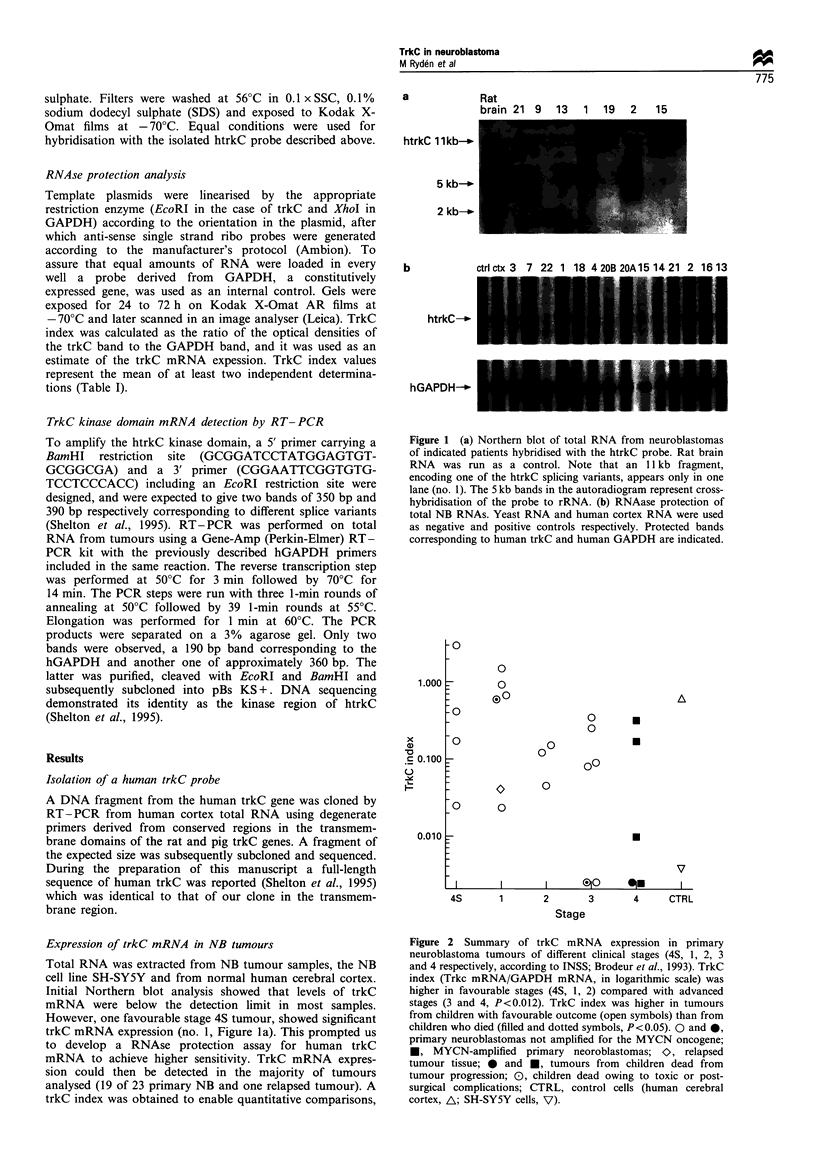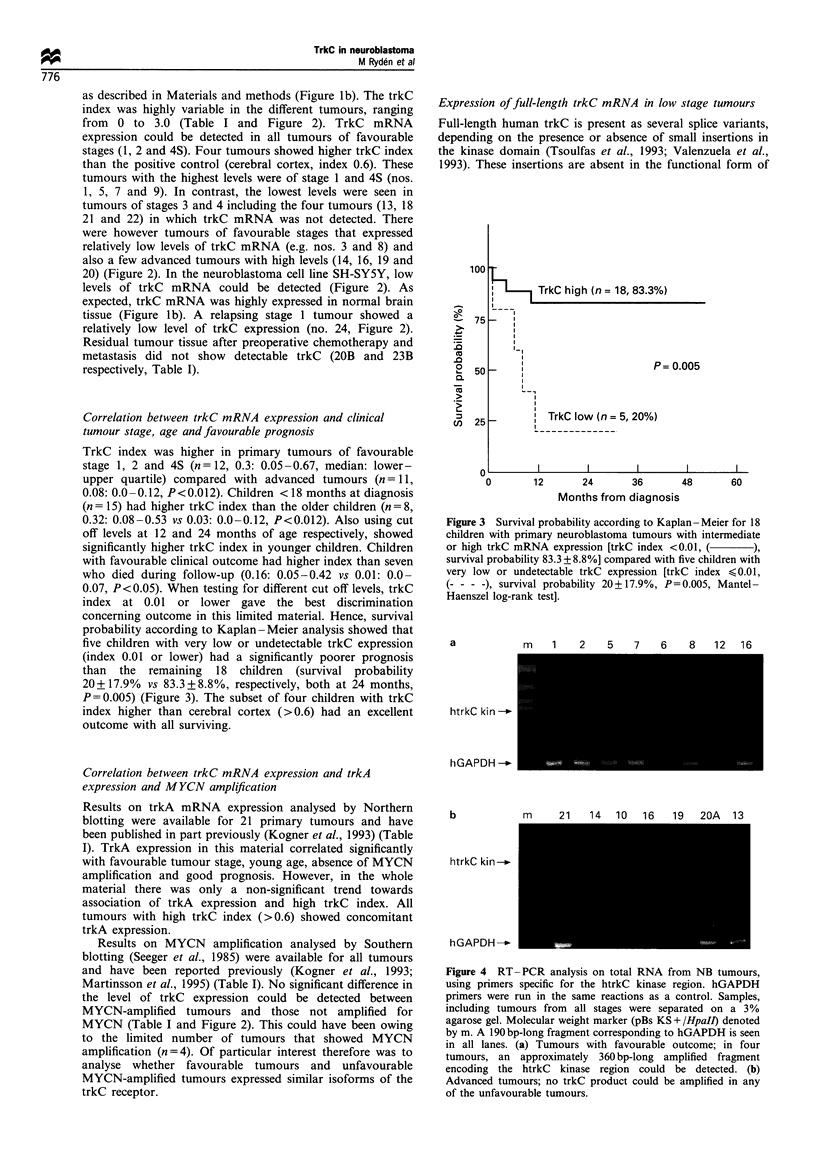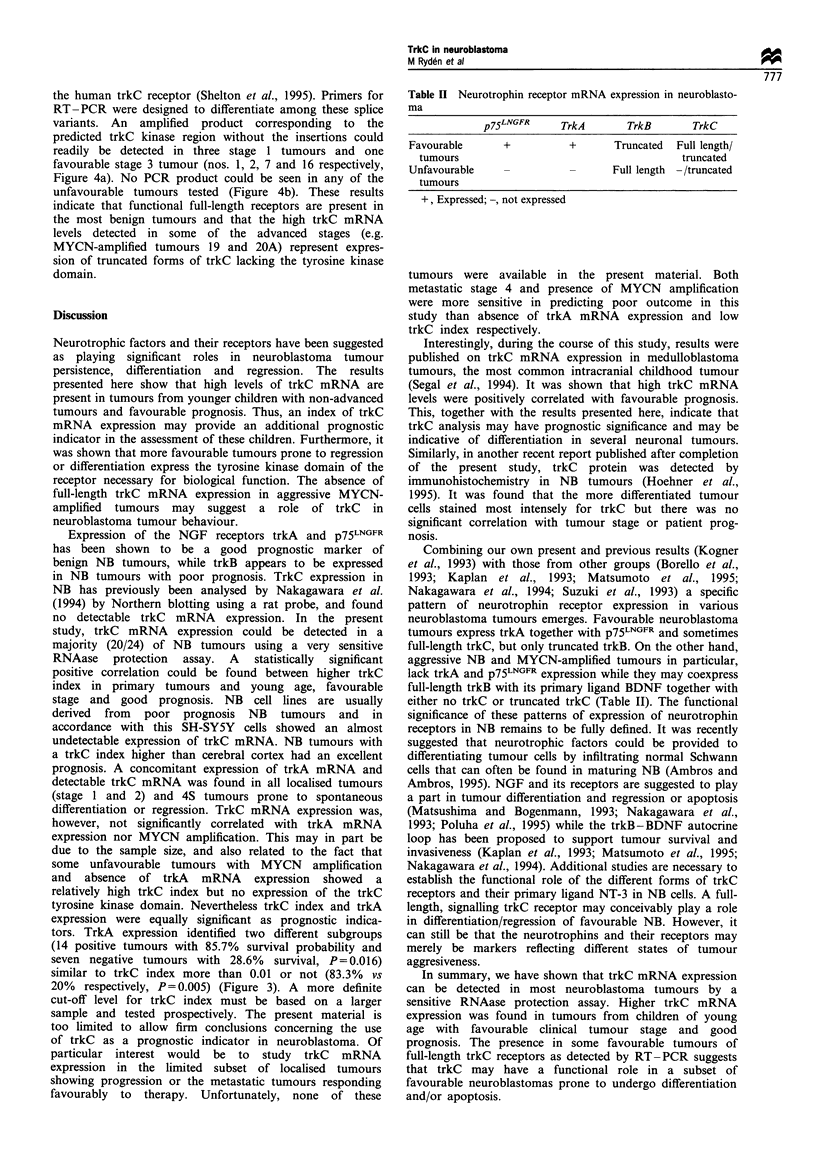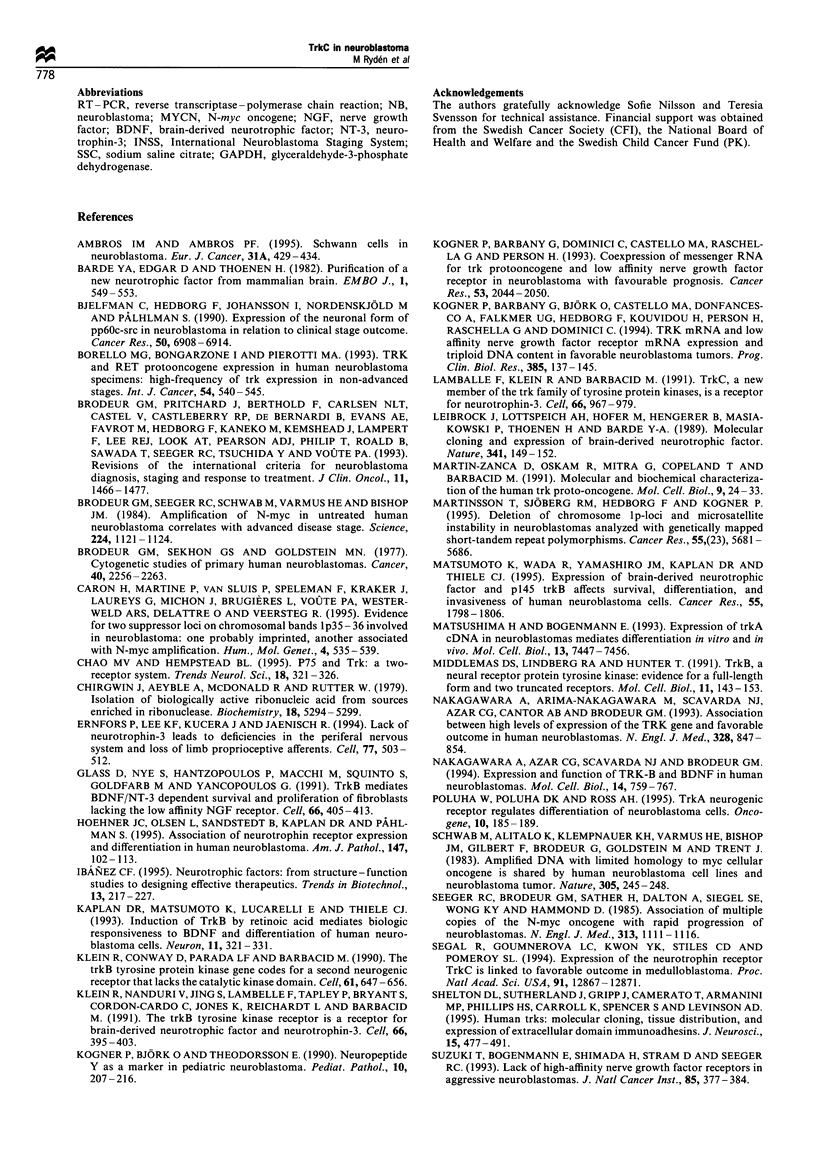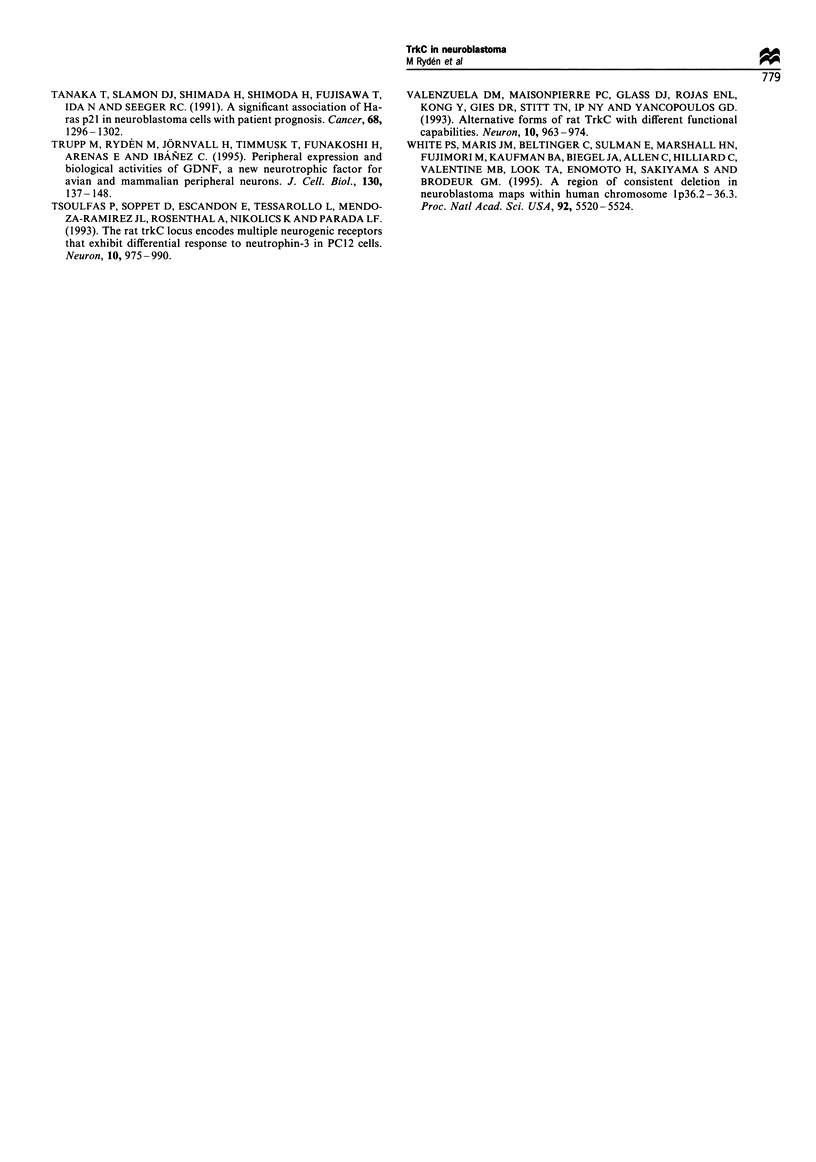# Expression of mRNA for the neurotrophin receptor trkC in neuroblastomas with favourable tumour stage and good prognosis.

**DOI:** 10.1038/bjc.1996.435

**Published:** 1996-09

**Authors:** M. Rydén, R. Sehgal, C. Dominici, F. H. Schilling, C. F. Ibáñez, P. Kogner

**Affiliations:** Department of Medical Biochemistry and Biophysics, Karolinska Institute, Stockholm, Sweden.

## Abstract

**Images:**


British Journal of Cancer (1996) 74, 773-779
© 1996 Stockton Press All rights reserved 0007-0920/96 $12.00

### Expression of mRNA for the neurotrophin receptor trkC in neuroblastomas with favourable tumour stage and good prognosis

M Rydén[1], R Sehgal[1,*], C Dominici[2], FH Schilling[3], CF Ibáñez[1] and P Kogner[4]

[1]*Laboratory of Molecular Neurobiology, Department of Medical Biochemistry and Biophysics, Karolinska Institute, Stockholm, Sweden;* [2]*Department of Pediatrics, University 'La Sapienza', Via Regina Elena 324, 00161 Rome, Italy;* [3]*Department of Pediatric Oncology-Hematology, Olga Hospital-Pediatric Center, Bismarckstraße 8, D-70176 Stuttgart 1, Germany;* [4]*Childhood Cancer Research Unit, Department of Woman and Child Health, and Department of Laboratory Medicine, Karolinska Institute, Karolinska Hospital, Stockholm, Sweden.*

**Summary** Childhood neuroblastoma tumours of the sympathetic nervous system show a remarkable clinical heterogeneity ranging from spontaneous regression to unfavourable outcome despite intensive therapy. Favourable neuroblastomas often express high levels of trkA mRNA, encoding the tyrosine kinase receptor for nerve growth factor. We have investigated mRNA expression for the neurotrophin receptor trkC in 23 primary neuroblastomas using a sensitive RNAase protection assay. TrkC expression was detected in 19 of these tumours at highly variable levels with a 300-fold difference between the highest and lowest values. Significantly higher levels of trkC mRNA were found in tumours from patients with favourable features such as low age ($P<0.012$), favourable tumour stage ($P<0.012$) and favourable prognosis ($P<0.05$). Children with intermediate or high trkC mRNA expression had better prognosis compared with those with low or undetectable levels (83.3% *vs* 20%, $P=0.005$). Further characterisation of trkC mRNA expression by reverse transcriptase-polymerase chain reaction (RT-PCR) showed that mRNA encoding the full-length cytoplasmic tyrosine kinase domain of the receptor was only expressed in a subset of favourable tumours. These data show that favourable neuroblastomas may express the full trkC receptor while advanced tumours, in particular MYCN-amplified neuroblastoma, seem to either express no trkC or truncated trkC receptors of as yet unknown biological function. These data are suggestive of a role for trkC and its preferred ligand neutotrophin-3, NT-3, in neuroblastoma differentiation and/or regression.

**Keywords:** childhood cancer; neurotrophin; trk; receptor tyrosine kinase

Neuroblastoma (NB) is a complex childhood tumour derived from sympathoadrenal progenitors of the neural crest with a broad clinical spectrum, including spontaneous regression of widespread disease and poor outcome despite highly intensive multimodal therapy. NB tumours are classified into localised (stage 1 and 2), regional (stage 3) and metastatic (stage 4) with an additional subset of widespread infant tumours (stage 4S), which often show spontaneous regression and/or differentiation (Brodeur *et al.*, 1993). Age at diagnosis is an important factor related to prognosis, the majority of neuroblastomas occurring under 1-2 years of age are of stages 1, 2 or 4S with a favourable clinical outcome. Patients over 1-2 years of age often present advanced tumours, and in this group survival rates have not improved significantly during recent years despite increasingly aggressive therapy. The clinical heterogeneity of NB has stimulated an intensive search for biological markers correlated with prognosis, in order to allow, at an early stage the assessment of the individual patient as having favourable or poor prognosis respectively. Genomic amplification of the N-*myc* oncogene (MYCN), is correlated with advanced stage and unfavourable outcome (Brodeur *et al.*, 1984; Schwab *et al.*, 1983; Seeger *et al.*, 1985). Genetic aberrations found in NB include other structural chromosomal abnormalities, in particular deletions involving the short arm of chromosome 1 (Brodeur *et al.*, 1977). Recent data indicate that chromosome 1p harbours one or several tumour-suppressor genes that may be specific for the development of unfavourable NB (Caron *et al.*, 1995; Martinsson *et al.*, 1995; White *et al.*, 1995).

Other biological markers of prognostic significance include neuropeptides (e.g. neuropeptide Y) (Kogner *et al.*, 1990), ras and src proteins (Bjelfman *et al.*, 1990; Tanaka *et al.*, 1991) and neurotrophic factors and their receptors. Of the receptors, the most promising ones are the nerve growth factor (NGF) receptors trkA and $p75^{LNGFR}$ (Borello *et al.*, 1993; Kogner *et al.*, 1993; Nakagawara *et al.*, 1993; Suzuki *et al.*, 1993). TrkA encodes a receptor with tyrosine kinase activity essential for NGF signal transduction and function while the role of $p75^{LNGFR}$ in NGF signalling is still not fully understood (Chao and Hempstead, 1995; Ibáñez, 1995). NGF, the first discovered member of the neurotrophin family, is essential for differentiation and survival of sympathetic neurons and it has been suggested that NGF may be important for differentiation and regression of tumours of neural crest origin (Kogner *et al.*, 1993). Introduction of a functional trkA cDNA into NB cell lines allowed differentiation after NGF treatment indicating that loss of functional NGF receptor expression may play an important role in the behaviour of advanced NB (Matsushima and Bogenmann, 1993; Poluha *et al.*, 1995). In culture of primary NB tumours expressing trkA, NGF induced terminal differentiation, whereas NGF deprivation resulted in cell death (Nakagawara *et al.*, 1993). In agreement with this, trkA expression has been found to be associated with young age at diagnosis and non-advanced stages of NB tumours. Simultaneous expression of trkA and $p75^{LNGFR}$ mRNA has been shown to define a favourable subset of NB tumours which either differentiate or regress spontaneously and/or respond to therapy (Kogner *et al.*, 1993; Nakagawara *et al.*, 1993). Analysis of trkA and p75 mRNA, combined with analysis of DNA content in tumour cells, may predict clinical outcome in most children with NB (Kogner *et al.*, 1994).

The role of NGF and its receptors in NB has raised interest in the possible participation of other members of the neurotrophin family. Brain-derived neurotrophic factor (BDNF) (Barde *et al.*, 1982; Leibrock *et al.*, 1989) and its preferred receptor trkB (Glass *et al.*, 1991; Klein *et al.*, 1991) have been shown to be expressed at high levels in many NB

Correspondence: CF Ibáñez, Laboratory of Molecular Neurobiology, Department of Medical Biochemistry and Biophysics, Karolinska Institute, S-171 77 Stockholm, Sweden
*Present address: Neuroscience Unit, Howard Hughes Medical Center, University of California, San Francisco, CA, USA
Received 13 November 1995; revised 26 February 1996; accepted 7 March 1996

tumours with poor prognosis (Kaplan *et al.*, 1993; Nakagawara *et al.*, 1994). *In vitro* studies on NB cell lines have shown that BDNF increases NB cell survival, neurite extension and stimulates invasiveness (Matsumoto *et al.*, 1995). Several tumours with amplification of MYCN and poor clinical prognosis also express BDNF which may constitute an autocrine or paracrine loop stimulating tumour growth. TrkB can be expressed either as a full-length receptor or in a truncated non-signalling form lacking the tyrosine kinase domain (Klein *et al.*, 1990; Middlemas *et al.*, 1991). Interestingly, maturing tumours appear more likely to express the truncated form of this receptor (Nakagawara *et al.*, 1994).

In this study we have investigated the mRNA expression profile of the third member of the neurotrophin receptor tyrosine kinase family, trkC, a receptor for neurotrophin-3 (NT-3) (Lamballe *et al.*, 1991) which is involved in the survival of subpopulations of neural crest and placode-derived sensory neurons (Ernfors *et al.*, 1994). Our results indicate that higher levels of trkC mRNA expression correlate significantly with localised disease stage, young age and good prognosis, suggesting that, like trkA, trkC could probably provide a useful marker in the clinical evaluation of NB tumours. In addition, trkC mRNA found in a few of the advanced unfavourable neuroblastomas was of the truncated subtype, i.e. lacking the intracellular tyrosine kinase domain. These data indicate that, although a few aggressive neuroblastomas may express trkC receptors, these are of the truncated types, suggesting a role for trkC signalling in neuroblastoma regression and/or differentiation.

**Table I** Patient material and trkC expression

| *Patient number* | *Stage*[a] | *Age (months)* | *Outcome* | *Follow-up (months)* | *TrkA*[b] | *MYCN*[c] | *TrkC index*[d] |
|---|---|---|---|---|---|---|---|
| 1 | 4S | 0 | NED[e] | 26+ | + | <3 | 3 |
| 2 | 4S | 0 | NED | 48+ | + | <3 | 0.42 |
| 3 | 4S | 5 | NED | 43+ | NA[f] | <3 | 0.025 |
| 4 | 4S | 5 | NED | 35+ | + | <3 | 0.17 |
| 5 | 1 | 0 | NED | 52+ | + | <3 | 0.93 |
| 6 | 1 | 1 | Dead[g] | 0.5 | + | <3 | 0.59 |
| 7 | 1 | 9 | NED | 29+ | + | <3 | 0.67 |
| 8 | 1 | 12 | NED | 36+ | + | <3 | 0.023 |
| 9 | 1 | 17 | NED | 27+ | + | <3 | 1.5 |
| 01 | 2A | 33 | NED | 19+ | + | <3 | 0.045 |
| 11 | 2A | 60 | NED | 21+ | NA | <3 | 0.15 |
| 12 | 2B | 31 | NED | 37+ | + | <3 | 0.12 |
| 13 | 3 | 0 | Dead | 0.5 | + | <3 | 0 |
| 14 | 3 | 2 | NED | 18+ | – | <3 | 0.25 |
| 15 | 3 | 6 | NED | 47+ | + | <3 | 0.08 |
| 16 | 3 | 7 | NED | 25+ | – | <3 | 0.34 |
| 17 | 3 | 10 | NED | 37+ | + | <3 | 0.09 |
| 18 | 3 | 11 | NED | 31+ | + | <3 | 0 |
| 19 | 4 | 10 | DOD[h] | 10 | – | >10 | 0.32 |
| 20A | 4 | 19 | DOD | 4 | – | >10 | 0.17 |
| 20B[i] | 4(res) | 19 | DOD | 4 | – | >10 | 0 |
| 21 | 4 | 30 | DOD | 11 | – | <3 | 0 |
| 22 | 4 | 50 | DOD | 6 | – | >10 | 0 |
| 23A | 4 | 137 | DOD | 8 | – | >10 | 0.01 |
| 23B[j] | 4 (met) | 137 | DOD | 8 | – | >10 | 0 |
| 24[k] | 1 (rel) | 30 | NED | 12+ | – | <3 | 0.04 |

[a]According to International Neuroblastoma Staging System (Broduer *et al.*, 1993). [b]Trka mRNA expression by Northern blot analysis (Kogner *et al.*, 1993). [c]MYCN gene copy number per haploid genome by Southern blot analysis (Seeger *et al.*, 1985). [d]TrkC index = TrkC mRNA/GAPDH mRNA. [e]NED, no evidence of disease. [f]NA, not analysed. [g]Dead, dead from toxic or post-surgical complications. [h]DOD, dead of disease. [i]20B, residual tumour tissue after chemotherapy. [j]23B, metastatic tissue. [k]Relapsed tumour tissue at primary site.

### Materials and methods

### *Patient material and sample handling*

Twenty-four children with neuroblastoma diagnosis according to International Neuroblastoma Staging System (INSS) criteria (Brodeur *et al.*, 1993) including tumours of all five clinical stages (4S, 1, 2, 3 and 4) were included in the study, based on availability of tumour tissue for trkC analysis (Table I). Primary tumour tissue was available from 23 children, while from one, only relapsing tumour tissue was analysed. In addition to primary tumours, a metastasis was analysed from one child, and from another, residual tumour tissue obtained after chemotherapy could be compared with primary tumour tissue removed before therapy. Data from the 23 children with primary tumours were used for calculation of correlations with clinical features and prognosis. Seven children died during follow-up after 0.5–11 months (median 6 months), five owing to tumour progression and two owing to toxic and/or surgical complications (Table I). Survival probability for the whole group of 23 children was 69.6% (standard error 9.6%). The 16 survivors were followed for 18–52 months (median 33 months). Tumour tissue from all patients was surgically resected and immediately frozen at −70°C until analysis. Tumour cell content of the samples was assessed histologically in tumour tissue adjacent to that used for RNA extraction.

### *Statistical calculations*

The Wilcoxon–Mann–Whitney rank sum test was used for the comparison of two independent samples. Survival probability was calculated using the Kaplan–Meier method and compared using the Mantel–Haenszel log-rank test.

### *DNA cloning*

A cDNA fragment encoding the transmembrane domain of human trkC (htrkC), was amplified by RT–PCR on human cortex total RNA using a 5′ rat derived primer (GGAATTCCATTTGGGGTATCCATAGCTG) and a 3′ porcine-derived primer (GGGAAGCTTCTTCCCAAAG-GCTCCCTCAC) yielding an approximately 380 bp-long fragment. It was subsequently subcloned into pBS KS+ (Stratagene). DNA sequencing revealed a sequence with significant homology to trkC sequences previously isolated from other species. A 190 bp-long cDNA fragment of the human glyceraldehyde-3-phosphate dehydrogenase (hGA-PDH) gene spanning bases 675 to 865 was amplified by RT–PCR on human cortex total RNA using a 5′ primer (CAGAATTCTGCCTCTACTGGCGCT) and a 3′ primer (CAGGATCCGACGCCTGCTTCACCA) and subcloned into pBS KS+.

### *RNA preparation*

Total RNA was prepared by homogenisation in 4 M guanidine isothiocyanate followed by either centrifugation through a caesium chloride cushion (Chirgwin *et al.*, 1979) or treatment with sodium acetate (pH 4.0) and a phenol–chloroform extraction (Trupp *et al.*, 1995) depending on the amount of tissue.

### *Northern blots*

Equal amounts (20 $\mu g$) of total RNA were separated in a denaturing 1% agarose gel containing 0.7% formaldehyde and 0.1 mg $ml^{-1}$ ethidium bromide. After electrophoresis the gels were examined under UV light to control for equal RNA loading in each sample. RNA was blotted onto nitrocellulose membranes (Hybond C-extra, Amersham) and hybridised to a 2.7 kb *Eco*RI fragment from a human trkA cDNA clone (Martin-Zanca *et al.*, 1991). Probes were labelled with $\alpha$-[$^{32}P$]-dCTP by random priming or nick translation to a specific activity of $10^9$ c.p.m. $\mu g^{-1}$. Hybridisation was performed overnight in 4 × sodium saline citrate (SSC)(1 × SSC is 0.15 M sodium chloride, 0.015 M sodium citrate pH 7.0), 40% formamide, 1 × Denhardt's solution and 10% dextran sulphate. Filters were washed at 56°C in 0.1×SSC, 0.1% sodium dodecyl sulphate (SDS) and exposed to Kodak X-Omat films at −70°C. Equal conditions were used for hybridisation with the isolated htrkC probe described above.

*RNAse protection analysis*

Template plasmids were linearised by the appropriate restriction enzyme (*Eco*RI in the case of trkC and *Xho*I in GAPDH) according to the orientation in the plasmid, after which anti-sense single strand ribo probes were generated according to the manufacturer's protocol (Ambion). To assure that equal amounts of RNA were loaded in every well a probe derived from GAPDH, a constitutively expressed gene, was used as an internal control. Gels were exposed for 24 to 72 h on Kodak X-Omat AR films at −70°C and later scanned in an image analyser (Leica). TrkC index was calculated as the ratio of the optical densities of the trkC band to the GAPDH band, and it was used as an estimate of the trkC mRNA expession. TrkC index values represent the mean of at least two independent determinations (Table I).

*TrkC kinase domain mRNA detection by RT–PCR*

To amplify the htrkC kinase domain, a 5′ primer carrying a *Bam*HI restriction site (GCGGATCCTATGGAGTGT-GCGGCGA) and a 3′ primer (CGGAATTCGGTGTG-TCCTCCCACC) including an *Eco*RI restriction site were designed, and were expected to give two bands of 350 bp and 390 bp respectively corresponding to different splice variants (Shelton *et al.*, 1995). RT–PCR was performed on total RNA from tumours using a Gene-Amp (Perkin-Elmer) RT–PCR kit with the previously described hGAPDH primers included in the same reaction. The reverse transcription step was performed at 50°C for 3 min followed by 70°C for 14 min. The PCR steps were run with three 1-min rounds of annealing at 50°C followed by 39 1-min rounds at 55°C. Elongation was performed for 1 min at 60°C. The PCR products were separated on a 3% agarose gel. Only two bands were observed, a 190 bp band corresponding to the hGAPDH and another one of approximately 360 bp. The latter was purified, cleaved with *Eco*RI and *Bam*HI and subsequently subcloned into pBs KS+. DNA sequencing demonstrated its identity as the kinase region of htrkC (Shelton *et al.*, 1995).

### Results

*Isolation of a human trkC probe*

A DNA fragment from the human trkC gene was cloned by RT–PCR from human cortex total RNA using degenerate primers derived from conserved regions in the transmembrane domains of the rat and pig trkC genes. A fragment of the expected size was subsequently subcloned and sequenced. During the preparation of this manuscript a full-length sequence of human trkC was reported (Shelton *et al.*, 1995) which was identical to that of our clone in the transmembrane region.

*Expression of trkC mRNA in NB tumours*

Total RNA was extracted from NB tumour samples, the NB cell line SH-SY5Y and from normal human cerebral cortex. Initial Northern blot analysis showed that levels of trkC mRNA were below the detection limit in most samples. However, one favourable stage 4S tumour, showed significant trkC mRNA expression (no. 1, Figure 1a). This prompted us to develop a RNAse protection assay for human trkC mRNA to achieve higher sensitivity. TrkC mRNA expression could then be detected in the majority of tumours analysed (19 of 23 primary NB and one relapsed tumour). A trkC index was obtained to enable quantitative comparisons,

**Figure 1** (**a**) Northern blot of total RNA from neuroblastomas of indicated patients hybridised with the htrkC probe. Rat brain RNA was run as a control. Note that an 11 kb fragment, encoding one of the htrkC splicing variants, appears only in one lane (no. 1). The 5 kb bands in the autoradiogram represent cross-hybridisation of the probe to rRNA. (**b**) RNAase protection of total NB RNAs. Yeast RNA and human cortex RNA were used as negative and positive controls respectively. Protected bands corresponding to human trkC and human GAPDH are indicated.

**Figure 2** Summary of trkC mRNA expression in primary neuroblastoma tumours of different clinical stages (4S, 1, 2, 3 and 4 respectively, according to INSS; Brodeur *et al.*, 1993). TrkC index (Trkc mRNA/GAPDH mRNA, in logarithmic scale) was higher in favourable stages (4S, 1, 2) compared with advanced stages (3 and 4, $P<0.012$). TrkC index was higher in tumours from children with favourable outcome (open symbols) than from children who died (filled and dotted symbols, $P<0.05$). ○ and ●, primary neuroblastomas not amplified for the MYCN oncogene; ■, MYCN-amplified primary neoroblastomas; ◇, relapsed tumour tissue; ● and ■, tumours from children dead from tumour progression; ⊙, children dead owing to toxic or post-surgical complications; CTRL, control cells (human cerebral cortex, △; SH-SY5Y cells, ▽).

as described in Materials and methods (Figure 1b). The trkC index was highly variable in the different tumours, ranging from 0 to 3.0 (Table I and Figure 2). TrkC mRNA expression could be detected in all tumours of favourable stages (1, 2 and 4S). Four tumours showed higher trkC index than the positive control (cerebral cortex, index 0.6). These tumours with the highest levels were of stage 1 and 4S (nos. 1, 5, 7 and 9). In contrast, the lowest levels were seen in tumours of stages 3 and 4 including the four tumours (13, 18 21 and 22) in which trkC mRNA was not detected. There were however tumours of favourable stages that expressed relatively low levels of trkC mRNA (e.g. nos. 3 and 8) and also a few advanced tumours with high levels (14, 16, 19 and 20) (Figure 2). In the neuroblastoma cell line SH-SY5Y, low levels of trkC mRNA could be detected (Figure 2). As expected, trkC mRNA was highly expressed in normal brain tissue (Figure 1b). A relapsing stage 1 tumour showed a relatively low level of trkC expression (no. 24, Figure 2). Residual tumour tissue after preoperative chemotherapy and metastasis did not show detectable trkC (20B and 23B respectively, Table I).

*Correlation between trkC mRNA expression and clinical tumour stage, age and favourable prognosis*

TrkC index was higher in primary tumours of favourable stage 1, 2 and 4S ($n=12$, 0.3: 0.05–0.67, median: lower–upper quartile) compared with advanced tumours ($n=11$, 0.08: 0.0–0.12, $P<0.012$). Children <18 months at diagnosis ($n=15$) had higher trkC index than the older children ($n=8$, 0.32: 0.08–0.53 *vs* 0.03: 0.0–0.12, $P<0.012$). Also using cut off levels at 12 and 24 months of age respectively, showed significantly higher trkC index in younger children. Children with favourable clinical outcome had higher index than seven who died during follow-up (0.16: 0.05–0.42 *vs* 0.01: 0.0–0.07, $P<0.05$). When testing for different cut off levels, trkC index at 0.01 or lower gave the best discrimination concerning outcome in this limited material. Hence, survival probability according to Kaplan–Meier analysis showed that five children with very low or undetectable trkC expression (index 0.01 or lower) had a significantly poorer prognosis than the remaining 18 children (survival probability $20\pm17.9\%$ *vs* $83.3\pm8.8\%$, respectively, both at 24 months, $P=0.005$) (Figure 3). The subset of four children with trkC index higher than cerebral cortex (>0.6) had an excellent outcome with all surviving.

*Correlation between trkC mRNA expression and trkA expression and MYCN amplification*

Results on trkA mRNA expression analysed by Northern blotting were available for 21 primary tumours and have been published in part previously (Kogner *et al.*, 1993) (Table I). TrkA expression in this material correlated significantly with favourable tumour stage, young age, absence of MYCN amplification and good prognosis. However, in the whole material there was only a non-significant trend towards association of trkA expression and high trkC index. All tumours with high trkC index (>0.6) showed concomitant trkA expression.

Results on MYCN amplification analysed by Southern blotting (Seeger *et al.*, 1985) were available for all tumours and have been reported previously (Kogner *et al.*, 1993; Martinsson *et al.*, 1995) (Table I). No significant difference in the level of trkC expression could be detected between MYCN-amplified tumours and those not amplified for MYCN (Table I and Figure 2). This could have been owing to the limited number of tumours that showed MYCN amplification ($n=4$). Of particular interest therefore was to analyse whether favourable tumours and unfavourable MYCN-amplified tumours expressed similar isoforms of the trkC receptor.

*Expression of full-length trkC mRNA in low stage tumours*

Full-length human trkC is present as several splice variants, depending on the presence or absence of small insertions in the kinase domain (Tsoulfas *et al.*, 1993; Valenzuela *et al.*, 1993). These insertions are absent in the functional form of

**Figure 3** Survival probability according to Kaplan–Meier for 18 children with primary neuroblastoma tumours with intermediate or high trkC mRNA expression [trkC index <0.01, (————), survival probability $83.3\pm8.8\%$] compared with five children with very low or undetectable trkC expression [trkC index ⩽0.01, (- - - -), survival probability $20\pm17.9\%$, $P=0.005$, Mantel–Haenszel log-rank test].

**Figure 4** RT–PCR analysis on total RNA from NB tumours, using primers specific for the htrkC kinase region. hGAPDH primers were run in the same reactions as a control. Samples, including tumours from all stages were separated on a 3% agarose gel. Molecular weight marker (pBs KS+/*HpaII*) denoted by m. A 190 bp-long fragment corresponding to hGAPDH is seen in all lanes. (**a**) Tumours with favourable outcome; in four tumours, an approximately 360 bp-long amplified fragment encoding the htrkC kinase region could be detected. (**b**) Advanced tumours; no trkC product could be amplified in any of the unfavourable tumours.

the human trkC receptor (Shelton *et al.*, 1995). Primers for RT–PCR were designed to differentiate among these splice variants. An amplified product corresponding to the predicted trkC kinase region without the insertions could readily be detected in three stage 1 tumours and one favourable stage 3 tumour (nos. 1, 2, 7 and 16 respectively, Figure 4a). No PCR product could be seen in any of the unfavourable tumours tested (Figure 4b). These results indicate that functional full-length receptors are present in the most benign tumours and that the high trkC mRNA levels detected in some of the advanced stages (e.g. MYCN-amplified tumours 19 and 20A) represent expression of truncated forms of trkC lacking the tyrosine kinase domain.

**Table II** Neurotrophin receptor mRNA expression in neuroblastoma

| | $p75^{LNGFR}$ | *TrkA* | *TrkB* | *TrkC* |
|---|---|---|---|---|
| Favourable tumours | + | + | Truncated | Full length/ truncated |
| Unfavourable tumours | – | – | Full length | –/truncated |

+, Expressed; –, not expressed

### Discussion

Neurotrophic factors and their receptors have been suggested as playing significant roles in neuroblastoma tumour persistence, differentiation and regression. The results presented here show that high levels of trkC mRNA are present in tumours from younger children with non-advanced tumours and favourable prognosis. Thus, an index of trkC mRNA expression may provide an additional prognostic indicator in the assessment of these children. Furthermore, it was shown that more favourable tumours prone to regression or differentiation express the tyrosine kinase domain of the receptor necessary for biological function. The absence of full-length trkC mRNA expression in aggressive MYCN-amplified tumours may suggest a role of trkC in neuroblastoma tumour behaviour.

Expression of the NGF receptors trkA and $p75^{LNGFR}$ has been shown to be a good prognostic marker of benign NB tumours, while trkB appears to be expressed in NB tumours with poor prognosis. TrkC expression in NB has previously been analysed by Nakagawara *et al.* (1994) by Northern blotting using a rat probe, and found no detectable trkC mRNA expression. In the present study, trkC mRNA expression could be detected in a majority (20/24) of NB tumours using a very sensitive RNAase protection assay. A statistically significant positive correlation could be found between higher trkC index in primary tumours and young age, favourable stage and good prognosis. NB cell lines are usually derived from poor prognosis NB tumours and in accordance with this SH-SY5Y cells showed an almost undetectable expression of trkC mRNA. NB tumours with a trkC index higher than cerebral cortex had an excellent prognosis. A concomitant expression of trkA mRNA and detectable trkC mRNA was found in all localised tumours (stage 1 and 2) and 4S tumours prone to spontaneous differentiation or regression. TrkC mRNA expression was, however, not significantly correlated with trkA mRNA expression nor MYCN amplification. This may in part be due to the sample size, and also related to the fact that some unfavourable tumours with MYCN amplification and absence of trkA mRNA expression showed a relatively high trkC index but no expression of the trkC tyrosine kinase domain. Nevertheless trkC index and trkA expression were equally significant as prognostic indicators. TrkA expression identified two different subgroups (14 positive tumours with 85.7% survival probability and seven negative tumours with 28.6% survival, $P=0.016$) similar to trkC index more than 0.01 or not (83.3% *vs* 20% respectively, $P=0.005$) (Figure 3). A more definite cut-off level for trkC index must be based on a larger sample and tested prospectively. The present material is too limited to allow firm conclusions concerning the use of trkC as a prognostic indicator in neuroblastoma. Of particular interest would be to study trkC mRNA expression in the limited subset of localised tumours showing progression or the metastatic tumours responding favourably to therapy. Unfortunately, none of these tumours were available in the present material. Both metastatic stage 4 and presence of MYCN amplification were more sensitive in predicting poor outcome in this study than absence of trkA mRNA expression and low trkC index respectively.

Interestingly, during the course of this study, results were published on trkC mRNA expression in medulloblastoma tumours, the most common intracranial childhood tumour (Segal *et al.*, 1994). It was shown that high trkC mRNA levels were positively correlated with favourable prognosis. This, together with the results presented here, indicate that trkC analysis may have prognostic significance and may be indicative of differentiation in several neuronal tumours. Similarly, in another recent report published after completion of the present study, trkC protein was detected by immunohistochemistry in NB tumours (Hoehner *et al.*, 1995). It was found that the more differentiated tumour cells stained most intensely for trkC but there was no significant correlation with tumour stage or patient prognosis.

Combining our own present and previous results (Kogner *et al.*, 1993) with those from other groups (Borello *et al.*, 1993; Kaplan *et al.*, 1993; Matsumoto *et al.*, 1995; Nakagawara *et al.*, 1994; Suzuki *et al.*, 1993) a specific pattern of neurotrophin receptor expression in various neuroblastoma tumours emerges. Favourable neuroblastoma tumours express trkA together with $p75^{LNGFR}$ and sometimes full-length trkC, but only truncated trkB. On the other hand, aggressive NB and MYCN-amplified tumours in particular, lack trkA and $p75^{LNGFR}$ expression while they may coexpress full-length trkB with its primary ligand BDNF together with either no trkC or truncated trkC (Table II). The functional significance of these patterns of expression of neurotrophin receptors in NB remains to be fully defined. It was recently suggested that neurotrophic factors could be provided to differentiating tumour cells by infiltrating normal Schwann cells that can often be found in maturing NB (Ambros and Ambros, 1995). NGF and its receptors are suggested to play a part in tumour differentiation and regression or apoptosis (Matsushima and Bogenmann, 1993; Nakagawara *et al.*, 1993; Poluha *et al.*, 1995) while the trkB–BDNF autocrine loop has been proposed to support tumour survival and invasiveness (Kaplan *et al.*, 1993; Matsumoto *et al.*, 1995; Nakagawara *et al.*, 1994). Additional studies are necessary to establish the functional role of the different forms of trkC receptors and their primary ligand NT-3 in NB cells. A full-length, signalling trkC receptor may conceivably play a role in differentiation/regression of favourable NB. However, it can still be that the neurotrophins and their receptors may merely be markers reflecting different states of tumour aggresiveness.

In summary, we have shown that trkC mRNA expression can be detected in most neuroblastoma tumours by a sensitive RNAase protection assay. Higher trkC mRNA expression was found in tumours from children of young age with favourable clinical tumour stage and good prognosis. The presence in some favourable tumours of full-length trkC receptors as detected by RT–PCR suggests that trkC may have a functional role in a subset of favourable neuroblastomas prone to undergo differentiation and/or apoptosis.

**Abbreviations**

RT-PCR, reverse transcriptase-polymerase chain reaction; NB, neuroblastoma; MYCN, N-*myc* oncogene; NGF, nerve growth factor; BDNF, brain-derived neurotrophic factor; NT-3, neurotrophin-3; INSS, International Neuroblastoma Staging System; SSC, sodium saline citrate; GAPDH, glyceraldehyde-3-phosphate dehydrogenase.

**Acknowledgements**

The authors gratefully acknowledge Sofie Nilsson and Teresia Svensson for technical assistance. Financial support was obtained from the Swedish Cancer Society (CFI), the National Board of Health and Welfare and the Swedish Child Cancer Fund (PK).

### References

AMBROS IM AND AMBROS PF. (1995). Schwann cells in neuroblastoma. *Eur. J. Cancer*, **31A**, 429-434.

BARDE YA, EDGAR D AND THOENEN H. (1982). Purification of a new neurotrophic factor from mammalian brain. *EMBO J.*, **1**, 549-553.

BJELFMAN C, HEDBORG F, JOHANSSON I, NORDENSKJÖLD M AND PÅLHLMAN S. (1990). Expression of the neuronal form of pp60c-src in neuroblastoma in relation to clinical stage outcome. *Cancer Res.*, **50**, 6908-6914.

BORELLO MG, BONGARZONE I AND PIEROTTI MA. (1993). TRK and RET protooncogene expression in human neuroblastoma specimens: high-frequency of trk expression in non-advanced stages. *Int. J. Cancer*, **54**, 540-545.

BRODEUR GM, PRITCHARD J, BERTHOLD F, CARLSEN NLT, CASTEL V, CASTLEBERRY RP, DE BERNARDI B, EVANS AE, FAVROT M, HEDBORG F, KANEKO M, KEMSHEAD J, LAMPERT F, LEE REJ, LOOK AT, PEARSON ADJ, PHILIP T, ROALD B, SAWADA T, SEEGER RC, TSUCHIDA Y AND VOÛTE PA. (1993). Revisions of the international criteria for neuroblastoma diagnosis, staging and response to treatment. *J Clin. Oncol.*, **11**, 1466-1477.

BRODEUR GM, SEEGER RC, SCHWAB M, VARMUS HE AND BISHOP JM. (1984). Amplification of N-myc in untreated human neuroblastoma correlates with advanced disease stage. *Science*, **224**, 1121-1124.

BRODEUR GM, SEKHON GS AND GOLDSTEIN MN. (1977). Cytogenetic studies of primary human neuroblastomas. *Cancer*, **40**, 2256-2263.

CARON H, MARTINE P, VAN SLUIS P, SPELEMAN F, KRAKER J, LAUREYS G, MICHON J, BRUGIÈRES L, VOÛTE PA, WESTERWELD ARS, DELATTRE O AND VEERSTEG R. (1995). Evidence for two suppressor loci on chromosomal bands 1p35-36 involved in neuroblastoma: one probably imprinted, another associated with N-myc amplification. *Hum., Mol. Genet.*, **4**, 535-539.

CHAO MV AND HEMPSTEAD BL. (1995). P75 and Trk: a two-receptor system. *Trends Neurol. Sci.*, **18**, 321-326.

CHIRGWIN J, AEYBLE A, MCDONALD R AND RUTTER W. (1979). Isolation of biologically active ribonucleic acid from sources enriched in ribonuclease. *Biochemistry*, **18**, 5294-5299.

ERNFORS P, LEE KF, KUCERA J AND JAENISCH R. (1994). Lack of neurotrophin-3 leads to deficiencies in the periferal nervous system and loss of limb proprioceptive afferents. *Cell*, **77**, 503-512.

GLASS D, NYE S, HANTZOPOULOS P, MACCHI M, SQUINTO S, GOLDFARB M AND YANCOPOULOS G. (1991). TrkB mediates BDNF/NT-3 dependent survival and proliferation of fibroblasts lacking the low affinity NGF receptor. *Cell*, **66**, 405-413.

HOEHNER JC, OLSEN L, SANDSTEDT B, KAPLAN DR AND PÅHLMAN S. (1995). Association of neurotrophin receptor expression and differentiation in human neuroblastoma. *Am. J. Pathol.*, **147**, 102-113.

IBÁÑEZ CF. (1995). Neurotrophic factors: from structure-function studies to designing effective therapeutics. *Trends in Biotechnol.*, **13**, 217-227.

KAPLAN DR, MATSUMOTO K, LUCARELLI E AND THIELE CJ. (1993). Induction of TrkB by retinoic acid mediates biologic responsiveness to BDNF and differentiation of human neuroblastoma cells. *Neuron*, **11**, 321-331.

KLEIN R, CONWAY D, PARADA LF AND BARBACID M. (1990). The trkB tyrosine protein kinase gene codes for a second neurogenic receptor that lacks the catalytic kinase domain. *Cell*, **61**, 647-656.

KLEIN R, NANDURI V, JING S, LAMBELLE F, TAPLEY P, BRYANT S, CORDON-CARDO C, JONES K, REICHARDT L AND BARBACID M. (1991). The trkB tyrosine kinase receptor is a receptor for brain-derived neurotrophic factor and neurotrophin-3. *Cell*, **66**, 395-403.

KOGNER P, BJÖRK O AND THEODORSSON E. (1990). Neuropeptide Y as a marker in pediatric neuroblastoma. *Pediat. Pathol.*, **10**, 207-216.

KOGNER P, BARBANY G, DOMINICI C, CASTELLO MA, RASCHELLA G AND PERSON H. (1993). Coexpression of messenger RNA for trk protooncogene and low affinity nerve growth factor receptor in neuroblastoma with favourable prognosis. *Cancer Res.*, **53**, 2044-2050.

KOGNER P, BARBANY G, BJÖRK O, CASTELLO MA, DONFANCESCO A, FALKMER UG, HEDBORG F, KOUVIDOU H, PERSON H, RASCHELLA G AND DOMINICI C. (1994). TRK mRNA and low affinity nerve growth factor receptor mRNA expression and triploid DNA content in favorable neuroblastoma tumors. *Prog. Clin. Biol. Res.*, **385**, 137-145.

LAMBALLE F, KLEIN R AND BARBACID M. (1991). TrkC, a new member of the trk family of tyrosine protein kinases, is a receptor for neurotrophin-3. *Cell*, **66**, 967-979.

LEIBROCK J, LOTTSPEICH AH, HOFER M, HENGERER B, MASIAKOWSKI P, THOENEN H AND BARDE Y-A. (1989). Molecular cloning and expression of brain-derived neurotrophic factor. *Nature*, **341**, 149-152.

MARTIN-ZANCA D, OSKAM R, MITRA G, COPELAND T AND BARBACID M. (1991). Molecular and biochemical characterization of the human trk proto-oncogene. *Mol. Cell. Biol.*, **9**, 24-33.

MARTINSSON T, SJÖBERG RM, HEDBORG F AND KOGNER P. (1995). Deletion of chromosome 1p-loci and microsatellite instability in neuroblastomas analyzed with genetically mapped short-tandem repeat polymorphisms. *Cancer Res.*, **55**,(23), 5681-5686.

MATSUMOTO K, WADA R, YAMASHIRO JM, KAPLAN DR AND THIELE CJ. (1995). Expression of brain-derived neurotrophic factor and p145 trkB affects survival, differentiation, and invasiveness of human neuroblastoma cells. *Cancer Res.*, **55**, 1798-1806.

MATSUSHIMA H AND BOGENMANN E. (1993). Expression of trkA cDNA in neuroblastomas mediates differentiation *in vitro* and *in vivo*. *Mol. Cell. Biol.*, **13**, 7447-7456.

MIDDLEMAS DS, LINDBERG RA AND HUNTER T. (1991). TrkB, a neural receptor protein tyrosine kinase: evidence for a full-length form and two truncated receptors. *Mol. Cell. Biol.*, **11**, 143-153.

NAKAGAWARA A, ARIMA-NAKAGAWARA M, SCAVARDA NJ, AZAR CG, CANTOR AB AND BRODEUR GM. (1993). Association between high levels of expression of the TRK gene and favorable outcome in human neuroblastomas. *N. Engl. J. Med.*, **328**, 847-854.

NAKAGAWARA A, AZAR CG, SCAVARDA NJ AND BRODEUR GM. (1994). Expression and function of TRK-B and BDNF in human neuroblastomas. *Mol. Cell. Biol.*, **14**, 759-767.

POLUHA W, POLUHA DK AND ROSS AH. (1995). TrkA neurogenic receptor regulates differentiation of neuroblastoma cells. *Oncogene*, **10**, 185-189.

SCHWAB M, ALITALO K, KLEMPNAUER KH, VARMUS HE, BISHOP JM, GILBERT F, BRODEUR G, GOLDSTEIN M AND TRENT J. (1983). Amplified DNA with limited homology to myc cellular oncogene is shared by human neuroblastoma cell lines and neuroblastoma tumor. *Nature*, **305**, 245-248.

SEEGER RC, BRODEUR GM, SATHER H, DALTON A, SIEGEL SE, WONG KY AND HAMMOND D. (1985). Association of multiple copies of the N-myc oncogene with rapid progression of neuroblastomas. *N. Engl. J. Med.*, **313**, 1111-1116.

SEGAL R, GOUMNEROVA LC, KWON YK, STILES CD AND POMEROY SL. (1994). Expression of the neurotrophin receptor TrkC is linked to favorable outcome in medulloblastoma. *Proc. Natl Acad. Sci. USA*, **91**, 12867-12871.

SHELTON DL, SUTHERLAND J, GRIPP J, CAMERATO T, ARMANINI MP, PHILLIPS HS, CARROLL K, SPENCER S AND LEVINSON AD. (1995). Human trks: molecular cloning, tissue distribution, and expression of extracellular domain immunoadhesins. *J. Neurosci.*, **15**, 477-491.

SUZUKI T, BOGENMANN E, SHIMADA H, STRAM D AND SEEGER RC. (1993). Lack of high-affinity nerve growth factor receptors in aggressive neuroblastomas. *J. Natl Cancer Inst.*, **85**, 377-384.

TANAKA T, SLAMON DJ, SHIMADA H, SHIMODA H, FUJISAWA T, IDA N AND SEEGER RC. (1991). A significant association of Ha-ras p21 in neuroblastoma cells with patient prognosis. *Cancer*, **68**, 1296–1302.

TRUPP M, RYDÉN M, JÖRNVALL H, TIMMUSK T, FUNAKOSHI H, ARENAS E AND IBÁÑEZ C. (1995). Peripheral expression and biological activities of GDNF, a new neurotrophic factor for avian and mammalian peripheral neurons. *J. Cell. Biol.*, **130**, 137–148.

TSOULFAS P, SOPPET D, ESCANDON E, TESSAROLLO L, MENDOZA-RAMIREZ JL, ROSENTHAL A, NIKOLICS K AND PARADA LF. (1993). The rat trkC locus encodes multiple neurogenic receptors that exhibit differential response to neutrophin-3 in PC12 cells. *Neuron*, **10**, 975–990.

VALENZUELA DM, MAISONPIERRE PC, GLASS DJ, ROJAS ENL, KONG Y, GIES DR, STITT TN, IP NY AND YANCOPOULOS GD. (1993). Alternative forms of rat TrkC with different functional capabilities. *Neuron*, **10**, 963–974.

WHITE PS, MARIS JM, BELTINGER C, SULMAN E, MARSHALL HN, FUJIMORI M, KAUFMAN BA, BIEGEL JA, ALLEN C, HILLIARD C, VALENTINE MB, LOOK TA, ENOMOTO H, SAKIYAMA S AND BRODEUR GM. (1995). A region of consistent deletion in neuroblastoma maps within human chromosome 1p36.2–36.3. *Proc. Natl Acad. Sci. USA*, **92**, 5520–5524.